# Supplementary material for: Complexome profiling of the Chlamydomonas psb28 mutant reveals TEF5 as an early PSII assembly factor
Source: Plant Cell. 2025 Mar 17;37(6):koaf055. doi: 10.1093/plcell/koaf055 (PMC12164590; doi:10.1093/plcell/koaf055)
Supplement: koaf055_Supplementary_Data [file koaf055_supplementary_data.zip › psb28_interactive.zip!/plots/intensity/qualityPlots/peptides/_Cre12.g558600.t1.2.html]

### Cre12.g558600.t1.2

Trivial name:   
SeqCoverage [median]: 24.20  
Mass: 50.09 kDa  
  
 

| Sequence | MissedCleavage | Mass [Da] | wtIntensity | muIntensity | Score |
| --- | --- | --- | --- | --- | --- |
| AAELLADAK | 0 | 900.49 | 1080.73 | 1735.18 | 120.15 |
| AAPLLEQALK | 0 | 1052.62 | 932.56 | 0.00 | 71.61 |
| APAPQAR | 0 | 709.39 | 0.00 | 479.99 | 74.83 |
| DASRETLTR | 1 | 1047.53 | 241.24 | 0.00 | 44.86 |
| GEQAGLVLVGSPSR | 0 | 1368.74 | 0.00 | 2123.01 | 115.78 |
| LHPNAVK | 0 | 777.45 | 0.00 | 333.57 | 80.38 |
| RLDAVLPAREEQGGAPAFR | 2 | 2052.09 | 0.00 | 858.12 | 29.27 |
| VLNDAVSEGGHLTQDGVPVVTHR | 0 | 2399.22 | 1795.61 | 1767.18 | 51.03 |
| VVTEEGNAEE | 0 | 1075.47 | 0.00 | 302.59 | 54.16 |
| YAAEAQQR | 0 | 935.45 | 213.68 | 0.00 | 65.04 |
|  |  |  | 4263.8 | 7599.6 |  |
